# Phenotypic Characterization of a Novel Virulence-Factor Deletion Strain of *Burkholderia mallei* That Provides Partial Protection against Inhalational Glanders in Mice

**DOI:** 10.3389/fcimb.2016.00021

**Published:** 2016-02-26

**Authors:** Joel A. Bozue, Sidhartha Chaudhury, Kei Amemiya, Jennifer Chua, Christopher K. Cote, Ronald G. Toothman, Jennifer L. Dankmeyer, Christopher P. Klimko, Catherine L. Wilhelmsen, Jolynn W. Raymond, Nela Zavaljevski, Jaques Reifman, Anders Wallqvist

**Affiliations:** ^1^Bacteriology Division, United States Army Medical Research Institute of Infectious DiseasesFort Detrick, MD, USA; ^2^Telemedicine and Advanced Technology Research Center, Biotechnology HPC Software Applications Institute, United States Army Medical Research and Materiel CommandFort Detrick, MD, USA; ^3^Pathology Division, United States Army of Medical Research Institute of Infectious DiseasesFort Detrick, MD, USA

**Keywords:** *Burkholderia mallei*, virulence factor, live-attenuated vaccine, glanders, aerosol

## Abstract

*Burkholderia mallei* (*Bm*) is a highly infectious intracellular pathogen classified as a category B biological agent by the Centers for Disease Control and Prevention. After respiratory exposure, *Bm* establishes itself within host macrophages before spreading into major organ systems, which can lead to chronic infection, sepsis, and death. Previously, we combined computational prediction of host-pathogen interactions with yeast two-hybrid experiments and identified novel virulence factor genes in *Bm*, including *BMAA0*553, *BMAA0728* (*tssN*), and *BMAA1865*. In the present study, we used recombinant allelic exchange to construct deletion mutants of *BMAA0553* and *tssN* (ΔBMAA0553 and ΔTssN, respectively) and showed that both deletions completely abrogated virulence at doses of >100 times the LD_50_ of the wild-type *Bm* strain. Analysis of ΔBMAA0553- and ΔTssN-infected mice showed starkly reduced bacterial dissemination relative to wild-type *Bm*, and subsequent *in vitro* experiments characterized pathogenic phenotypes with respect to intracellular growth, macrophage uptake and phagosomal escape, actin-based motility, and multinucleated giant cell formation. Based on observed *in vitro* and *in vivo* phenotypes, we explored the use of ΔTssN as a candidate live-attenuated vaccine. Mice immunized with aerosolized ΔTssN showed a 21-day survival rate of 67% after a high-dose aerosol challenge with the wild-type *Bm* ATCC 23344 strain, compared to a 0% survival rate for unvaccinated mice. However, analysis of histopathology and bacterial burden showed that while the surviving vaccinated mice were protected from acute infection, *Bm* was still able to establish a chronic infection. Vaccinated mice showed a modest IgG response, suggesting a limited potential of ΔTssN as a vaccine candidate, but also showed prolonged elevation of pro-inflammatory cytokines, underscoring the role of cellular and innate immunity in mitigating acute infection in inhalational glanders.

## Introduction

*Burkholderia mallei* (*Bm*) is the etiological agent of glanders (Boerner, 1882) and is classified as a Category B biological agent by the Centers for Disease Control and Prevention. Horses, donkeys, and mules are considered the natural host for *Bm* infection and serve as reservoirs for transmitting the disease to other animals and humans (Sharrer, 1995). Human infection, although rare, is almost always fatal without antibiotic intervention. While the most likely route of infection for *Bm* is contact with mucus discharge of an infected animal, *Bm* is also highly infectious by aerosol (Howe and Miller, 1947) and is considered to have potential use as a biological weapon (CDC Strategic Planning Workgroup, 2000). *Bm* infections have been shown to be highly resistant to antibiotics, and there is currently no approved vaccine for glanders.

*Bm* is a facultative intracellular pathogen that establishes itself in host macrophages after either respiratory or subcutaneous exposure (Howe and Miller, 1947; Srinivasan et al., 2001), resulting in an acute infection, followed by dissemination to major organ systems, chronic infection, sepsis, and death. *Bm* is equipped with an extensive set of mechanisms for invasion and modulation of host cell environments. Key mechanisms of *Bm* pathogenicity are encoded in virulence factors, proteins that control and promote pathogenic internalization, survival, and replication within host cells (Galyov et al., 2010; Allwood et al., 2011). A number of virulence factors have been characterized and play roles in adhesion, endosomal escape, evasion of host cell autophagy, actin-based motility, multinucleated giant cell (MNGC) formation, and replication (Nierman et al., 2004; Stevens et al., 2005a; Schell et al., 2007; Galyov et al., 2010; Losada et al., 2010; Lazar Adler et al., 2011; Memiševic et al., 2013).

In previous studies, we used a combined computational and experimental strategy to systematically identify and characterize the interaction between *Bm* virulence factors and their host targets (Memiševic et al., 2013, 2015). We used several bioinformatics approaches to select a small number of putative and known virulence factors and used yeast two-hybrid assays to determine their interacting partners in human and murine hosts. The analysis of these host-pathogen interactions allowed us to identify three novel *Bm* virulence factor genes, *BMAA0553, BMAA0728* (*tssN*), and *BMAA1865*, and show that insertion mutants generated for each of the three virulence factors attenuated *Bm* in mouse aerosol challenge experiments (Memiševic et al., 2013). *tssN* has been shown to be part of a gene cluster that encodes components of a type 6 secretion system (Schell et al., 2007), while little is known about *BMAA0553* or *BMAA1865*. Beyond showing virulence attenuation when disrupted, none of these three genes have been experimentally characterized. However, bioinformatics analyses using host-pathogen interaction data indicate that the *tssN* plays a role in ubiquitination and intracellular signaling, *BMAA0553* encodes a putative serine/threonine phosphatase that is involved in signaling, adhesion, and actin cytoskeletal rearrangement, and that *BMAA1865* plays a role in ubiquitination and phagosomal escape (Memiševic et al., 2014, 2015).

Attenuated strains can be used as live-attenuated vaccines (LAVs) that establish mild infections in the host, mimicking the infection of fully virulent wild-type (WT) strains and presenting appropriate antigens to the host immune system to induce durable antibody and cell-mediated immune responses (Drabner and Guzman, 2001). LAV candidates for both *Bm* (Ulrich et al., 2005; Mott et al., 2015) and the closely related *Burkholderia pseudomallei* (*Bp*) (Breitbach et al., 2008; Norris et al., 2011; Silva et al., 2013) are among the most promising *Burkholderia* vaccines to date; although some have demonstrated protection against acute infection, all consistently failed to completely prevent the establishment of chronic infection in the BALB/c mouse model (Ekström et al., 2013). Unimmunized BALB/c mice challenged by a lethal dose of *Bm* die within several days of exposure. LAV immunization can extend their survival beyond the acute stage of the infection, but 100% mortality is observed by 60 days. Finally, unlike naturally occurring or laboratory-passaged attenuated strains, genetically engineered strains can have well-defined mechanisms of attenuation, making them safer, better-characterized alternatives as candidate LAVs (Bozue et al., 2012).

As an extension of our previous study (Memiševic et al., 2013), we sought to use recombinant allelic exchange to engineer *Bm* deletion mutants for the virulence factors *BMAA0553, tssN*, and *BMAA1865* to characterize pathogenic phenotypes and explore their potential as LAVs. The insertion mutants generated previously (Memiševic et al., 2013) have two drawbacks: (1) the observed attenuation could have resulted from unintended disruption of genes downstream of the target gene and (2) the potential for the insertion mutant to revert to wild-type, limiting its potential as a candidate vaccine. Here, we successfully engineered in-frame deletion mutants for *BMAA0553* and *tssN* (termed ΔBMAA0553 and ΔTssN, respectively). We assessed the pathogenicity of ΔBMAA0553 and ΔTssN after aerosol exposure in BALB/c mice, characterized pathogenic phenotypes in *in vitro* infection models, evaluated the efficacy of ΔTssN as an LAV, and identified immune responses that may be associated with protection against glanders.

## Materials and methods

### Bacterial strains and media

The *Escherichia coli* and *Bm* strains and plasmids used in this study are shown in Supplementary Table 1. All *E. coli* strains were grown at 37°C on Luria-Bertani (LB) Lennox medium. *Bm* was grown on sheep blood agar plates or LB Lennox broth agar plates containing 4% glycerol. As indicated, antibiotics were used at the following concentrations: ampicillin, 50 μg/ml; kanamycin, 50 μg/ml; and polymyxin B, 15 μg/ml. After conjugal mating and animal challenges, we performed screening on *Burkholderia cepacia* agar (R01709, Remel Inc., Lenexa, KS) to confirm the presence of *Bm*. We performed this work at the United States Army Medical Research Institute of Infectious Diseases (USAMRIID), which is compliant with all Federal and Department of Defense regulations pertaining to the use of Select Agents.

### Construction of ΔBMAA0553 and ΔTssN mutants

To delete the *BMAA0553* gene from *Bm*, we initially tried to use PCR to amplify *BMAA0553* from *Bm* genomic DNA but had difficulty producing the correct PCR product. Instead, we resorted to using the homologous gene, *BPSS0403*, from the K96243 strain of *Bp*. The DNA fragment containing *BPSS0403* and its flanking region has 100% sequence identity to *BMAA0553*. The gene and flanking DNA was amplified from genomic DNA using Taq DNA polymerase and GC buffer (Qiagen Inc., Gaithersburg, MD) and cloned into the pCR2.1 plasmid. The resulting plasmid was digested using NruII, which removed an internal 546-bp fragment from the gene, and then re-ligated. The excised fragment containing the remaining 429-bp of the upstream and 486-bp of the downstream gene was then cloned into the NheI site of pMo130 (Hamad et al., 2009).

To delete *tssN*, the DNA fragment containing the gene and the flanking DNA was amplified using PCR from *Bm* genomic DNA. We ligated the PCR product into pWSK30 (Wang and Kushner, 1991) and removed the *tssN* gene from the plasmid through inverse PCR, in frame, retaining the first and last three nucleotides of the gene. We cloned the fragment containing the deletion into pJET2.1 (Fermentas Inc., Glen Burnie, MD) and then subcloned it into pMo130.

We attempted to carry out an in-frame deletion of *BMAA1865* but were unable to do so due to the presence of a 21-bp repeat sequence that is repeated 37 times upstream of *BMAA1865*. This repeat prevented PCR amplification of this region and construction of an in-frame deletion plasmid.

The pMo130-Δ*tssN* and pMo130-Δ*BMAA0553* plasmids were transformed into *E. coli* strain S17-1 (Simon et al., 1983) and mobilized via conjugation into *Bm* GRS 23344, a Δ*sacB* deletion mutant derived from ATCC 23344 (Schell et al., 2008). We performed the mating procedure to initially select for cointegrates as previously described (DeShazer et al., 2001). To select for allelic exchange mutants, we grew the *Bm* cointegrate strain in yeast-extract tryptone (YT) broth medium and then serially diluted it onto YT agar plates +5% sucrose. We screened colonies that grew in the presence of sucrose for the loss of kanamycin resistance (present on pMo130), and verified the deletions by PCR. All primers used in this study are detailed in Supplementary Table 2.

### Extracellular and intracellular growth assays

To examine growth in media, bacterial strains were grown overnight in LB, LB + 4% glycerol, or M9 minimal medium (supplemented with glucose at final concentration of 0.2%) and re-suspended in fresh broth to approximately equal optical density (OD). We established growth as an increase in the absorbance at an OD 600 nm (OD_600_) using an Infinite M200 pro (Tecan, Männedorf, Switzerland) microplate reader in 96-well microtiter plates. We analyzed quadruplicate samples and included uninoculated controls to confirm sterility.

For intracellular growth assays, we grew *Bm* in LB Lennox broth + 4% glycerol for 15–18 h. RAW 264.7 cells were maintained in DMEM with 6 mM L-glutamine and 10% fetal bovine serum at 37°C with 5% CO_2_. To study intracellular replication, we plated 2 × 10^5^ RAW 264.7 cells into 24-well tissue culture plates and cultured them for 2 days. The cells were then infected at the multiplicity of infection (MOI) of less than 10 bacteria per macrophage. After 1 h, cells were washed twice with PBS and then reincubated in cell culture media with 200 μg/ml gentamycin. At the times indicated, macrophage cells were lysed with 0.2% Triton X-100, serially diluted, and plated onto agar plates for colony forming unit (CFU) counts.

### Immunofluorescence staining and confocal microscopy

To examine phagosomal escape and actin tail formation, we seeded 2 × 10^5^ RAW 264.7 cells on a 12-mm no. 1-1/2 coverslip (Electron Microscopy Sciences, Hatfield, PA) and infected them at a MOI of 20 the following day. For MNGC formation experiments, we seeded 4 × 10^5^ cells onto coverslips and infected them at a MOI of 5 the following day. To remove extracellular bacteria, coverslips were washed vigorously with PBS after the 1 h infection. Coverslips were placed in antibiotic-free medium and further incubated for the times indicated. Cells were fixed with 4% paraformaldehyde and then blocked in PBS containing 0.25% saponin, 0.2% Bovine Serum Albumin (BSA) fraction V, and 0.2% fish skin gelatin (Sigma-Aldrich, St. Louis, MO) for 2 h. Cells were subsequently incubated with rabbit anti-*Burkholderia* antibody (1:1000) (D. Waag, USAMRIID) and/or rat anti-lysosomal associated membrane protein 1 (LAMP-1) (1:200) antibody (ID4B; Developmental Studies Hybridoma Bank, Iowa City, IA) overnight at 4°C. After being washed, cells were incubated with secondary antibody conjugated to a fluorophore (1:600, Jackson Immunoresearch, West Grove, PA) for 5 h. Cells were also stained with phalloidin and/or 4′,6-diamidino-2-phenylindole (DAPI, Life Technologies, Grand Island, NY) for 2 h before the coverslip was mounted with Fluoromount G (Electron Microscopy Sciences, Hatfield, PA). Separate sets of coverslips were used to score for LAMP-1 and actin localization. Confocal microscopy was performed on the Zeiss 700 Laser Scanning Microscopy System using Zen-Black Edition 2011 software (Carl Zeiss Microimaging, Thornwood, NY). MNGC images were collected using the × 40 (numerical aperture: 1.3) oil objective lens, whereas the × 100 (numerical aperture: 1.4) oil objective lens was used for phagosomal escape and actin tail experiments. The pinhole was set to ~1 Airy unit.

### Animal challenges

For all challenges, we prepared a suspension of *Bm* using LB Lennox broth + 4% glycerol. Broth cultures were grown overnight in a 37°C shaker at 150 rpm and adjusted for various challenge doses as determined by serial dilutions and plating. Female BALB/c mice (7–9 week old), obtained from the National Cancer Institute, were anesthetized via an intramuscular injection with ketamine, acepromazine, and xylazine. We carried out intranasal challenge for *Bm* strains ATCC 23344 and GRS 23344 by intranasal instillation with 50 μl of *Bm* suspended in PBS to compare the virulence of the two strains. We administered aerosolized doses of ATCC 23344, GRS 23344, ΔBMAA0553, and ΔTssN using a dynamic 30-liter humidity-controlled Plexiglas whole body exposure chamber, as previously described (Roy et al., 2003). We obtained an estimate of the inhaled doses as previously described (Glynn et al., 2005; Dabisch et al., 2012a,b). For all challenge experiments, mice were monitored several times each day, and mortality rates (or euthanasia when moribund) were recorded.

Aerosol-challenged mice (*n* = 5) were euthanized within a CO_2_ chamber at the specified time postinfection for *Bm* dissemination experiments. The lungs and spleens were harvested, rinsed, weighed, and then homogenized in 1 ml PBS using a tissue grinder (Kendall Healthcare Precision Disposable Tissue Grinder Systems, Covidien, Mansfield, MA). Homogenates were then serially diluted and plated on to sheep blood agar plates to determine CFU recovery.

### Vaccination experiments

Blood was collected from all mice via submandibular bleeding before the initiation of the experiments to obtain pre-immune sera. Mice were split into the following groups: a naïve, untreated control group (*n* = 10) and a vaccination group (*n* = 39). Vaccinated mice received a primary aerosol immunization of ΔTssN at day 0 followed by boost aerosol immunizations at day 21. Vaccinated mice (*n* = 15) at day 42, along with naïve control mice, were challenged with an aerosolized dose of the fully virulent *Bm* strain ATCC 23344 (4.3 × 10^4^ CFUs, or 43 LD_50_) and followed for 21 days to compare survival and time to death between groups. In order to monitor the bacterial dissemination, pathology, and immune responses induced by ΔTssN immunization, a subset of vaccinated mice (*n* = 5) were euthanized by CO_2_ exposure for pathology and CFU detection from various organs and a second subset of vaccinated mice (*n* = 5) were euthanized by exsanguination under deep anesthesia to obtain sera for testing antibody levels and spleens to perform splenocyte stimulation assays following each of the three aerosol immunizations.

Animal research at the United States Army Medical Research Institute of Infectious Diseases (USAMRIID) was conducted under an animal use protocol approved by the USAMRIID Institutional Animal Care and Use Committee in compliance with the Animal Welfare Act, Public Health Service Policy, and other federal statutes and regulations relating to animals and experiments involving animals. The facility where this research was conducted is accredited by the Association for Assessment and Accreditation of Laboratory Animal Care, International and adheres to principles stated in the 8th Edition of the Guide for the Care and Use of Laboratory Animals, National Research Council, 2011.

### Immunological assays

#### Antibody assay

The antibody titers in vaccinated mice were determined as previously described (Amemiya et al., 2002). Briefly, IgG, IgG1, and IgG2a titers in sera were determined by an ELISA using 96-well plates (Immulon 2 HB, round bottom, Fisher Scientific, Pittsburgh, PA). Irradiated *Bm* strains were diluted in 0.1 M carbonate buffer (pH 9.5) to a concentration of 10 μg/ml. Then, 50 μl of the diluted bacteria was placed into wells, and plates were incubated at 4°C overnight. Plates were washed with washing solution (1 × PBS and 0.05% Tween 20) and incubated with 100 μl of blocking solution (washing solution with 1% BSA) for 1 h at 37°C. Two-fold dilutions of mouse sera were made with blocking solution in triplicate, and plates were incubated for 1 h at 37°C. After the plates had been washed, 50 μl of 1:5000-diluted anti-Ig-horseradish peroxidase conjugate for IgG, IgG1, or IgG2a (Southern Biotechnology Associates, Inc., Birmingham, AL) were added to each well, and plates were incubated for 1 h at 37°C. After the plates were washed, 50 μl of a buffered H_2_O_2_ and 3,3′,5,5′-tetramethylbenzidine solution (BD Pharmingen, San Jose, CA) was added to each well, and plates were incubated for 15 min at room temperature. The reaction was stopped with 25 μl of 2 N sulfuric acid, and the amount of bound antibody was determined colorimetrically at 450 nm with a reference filter of 570 nm. Results are reported as the reciprocal of the highest dilution giving a mean OD of at least 0.1.

#### Spleen cell preparation

Splenocytes were used for cytokine/chemokine determination directly from the supernatant in the spleen extracts and after *in vitro* stimulation with irradiation inactivated *Bm*. Splenocytes were prepared as previously described (Amemiya et al., 2006). Briefly, spleens were excised from mice and disaggregated in wash medium (RPMI 1640 medium, Life Technologies, Grand Island, NY) with 25 mM HEPES, 2 mM glutamine, 100 U/ml penicillin and 100 μg/ml streptomycin to make the spleen extract. Aliquots of the spleen extract were stored at −70°C and irradiated before cytokine/chemokine determination.

To isolate splenocytes for *in vitro* stimulation, red blood cells in the remaining spleen extract were lysed with ACK Lysing buffer (BioWhittaker, Walkersville, MD) for 5 min and diluted with wash medium. Splenocytes were pelleted by centrifugation at 1200 rpm for 10 min, washed once with complete medium [wash medium containing 10% heat-inactivated fetal calf serum (GIBCO, Life Technology, Waltham, MA), 1 mM sodium pyruvate, 0.1 mM non-essential amino acids, and 50 μM 2-mercaptoethanol], and splenocytes were suspended in complete medium and counted.

Stimulation of splenocytes was carried out in 48-well plates (Costar 3548, Fisher Scientific) with 2 × 10^6^ cells/well in a final volume of 0.5 ml. Splenocytes were stimulated with 2.0 × 10^7^ irradiation-inactivated *Bm* (ATCC 23344) in complete medium for 45–48 h at 37°C and 5% CO_2_. Phorbol 12-myristate 13-acetate (PMA; Sigma, St. Louis, MO) with ionomycin (Sigma) at 40 and 500 ng/ml, respectively, were used as a positive control.

#### Cytokine analysis

Cytokine analysis was done on both the supernatant from the spleen extracts and on the supernatant from the *in vitro* stimulation of splenocytes. In both cases, samples were centrifuged at 1200 rpm for 10 min, and supernatants were collected and stored at -70°C until irradiation. Protein levels of cytokine/chemokines in the supernatants were measured by Luminex Mag Pix (Life Technologies, Waltham, MA) as per the manufacturer's protocol. The levels of the following 20 cytokines/chemokines in either spleen extract supernatant or in the *in vitro* stimulated splenocyte supernatant were measured: FGFb, GM-CSF, IFNγ, IL-1α, IL-1β, IL-2, IL-4, IL-5, IL-6, IL-10, IL-12, IL-13, IL-17, IP-10, KC, MCP-1, MIG, MIP-1α, TNFα, and VEGF.

### Pathology

Postmortem tissues collected from mice were routinely processed as previously described. Briefly, tissues were fixed in 10% neutral buffered formalin, embedded in paraffin, and sectioned for hematoxylin and eosin (HE) staining. Slides were analyzed at × 200 magnification.

### Statistics

Statistical significance was determined by an unpaired two-tailed Student's *t*-test to compare data of intracellular growth, phagosomal escape, actin tail formation, CFU recovery and localization experiments from macrophage experiments, and time-to-death (TTD) and cytokine expression following infection in mouse experiments, statistical significance. Fifty percent lethal dose (LD_50_) analysis was determined by a Bayesian probit analysis. Survival rates were compared between groups by Fisher exact tests with permutation adjustment for multiple comparisons using SAS (version 8.2, SAS Institute Inc., SAS OnlineDoc, Cary, NC).

## Results

### Mutant construction

We deleted the *BMAA0553* and *tssN* genes, in frame, using double recombination. In order to select for the desired recombinant, we used *Bm* GRS 23344 (Δ*sacB* sucrose-resistant derivative strain of ATCC 23344) as the background strain for generating mutants utilizing pMo130, a shuttle vector, which contains the *sacB* gene to select for strains that had resolved the plasmid in the presence of sucrose. Previous work has demonstrated that the GRS 23344 strain is as virulent as ATCC 23344, in *Galleria mellonella* larvae [3]. To further demonstrate that GRS 23344 is an appropriate background strain for virulence studies, we compared ATCC 23344 and GRS 23344 strains *in vitro* in various types of media and an *in vivo* murine pneumonic model of glanders. Growth curves using both a rich (LB) or minimal (M9) media showed little or no difference between ATCC 23344 and GRS 23344 (data not shown). LD_50_ values for both strains were also similar after intranasal challenge of BALB/c mice (1355 CFU for ATCC 23344 and 1868 CFU for GRS 23344). In addition, no significant difference between strains was noted in survival TTD values observed (Supplementary Table 3) except for the lowest challenge dose group. However, the *p*-value for this group was based upon the comparison in TTD for two mice that succumbed to infection with ATCC 23344 and one mouse that succumbed to challenge with GRS 23344.

To select for deletion mutants of *BMAA0553* or *tssN* in GRS 23344, strains that had undergone allelic exchange and excised the plasmid (containing *sacB*) were selected for by growth in the presence of sucrose. To determine if sucrose-resistant clones carried the deletion, colonies were screened for the appropriate gene deletion by PCR with the respective primer pairs. We observed the full-length PCR product for *tssN* of ~3 kb, as well as a 300-bp shift for those clones in which the gene was deleted (data not shown). Similarly, for *BMAA0553* deletion construction, a 4-kb band was observed for the full-length PCR product, whereas a 1.5-kb band shift was detected for those strains containing the gene deletion (data not shown).

We attempted to complement the two mutations by cloning the respective functional gene including upstream sequence into pBHR2 (Schell et al., 2007). The *E. coli* strains carrying the plasmids and respective functional genes were then transformed into ΔTssN or ΔBMAA0553 mutant strains via conjugation as described above. Kanamycin resistant clones were selected and the presence of the plasmid was verified by plasmid purification. However, the mutant strains transformed with the plasmid containing the functional gene showed abnormal structural morphology where they were much more elongated/bacillus in shape as compared to the parent or mutant strains (data not shown). These transformed strains were confirmed to be *Burkholderia* by growth on *Burkholderia cepacia* agar and reacted with a *Burkholderia* antibody (data not shown).

### Extracellular and intracellular growth

**Figure 1:**
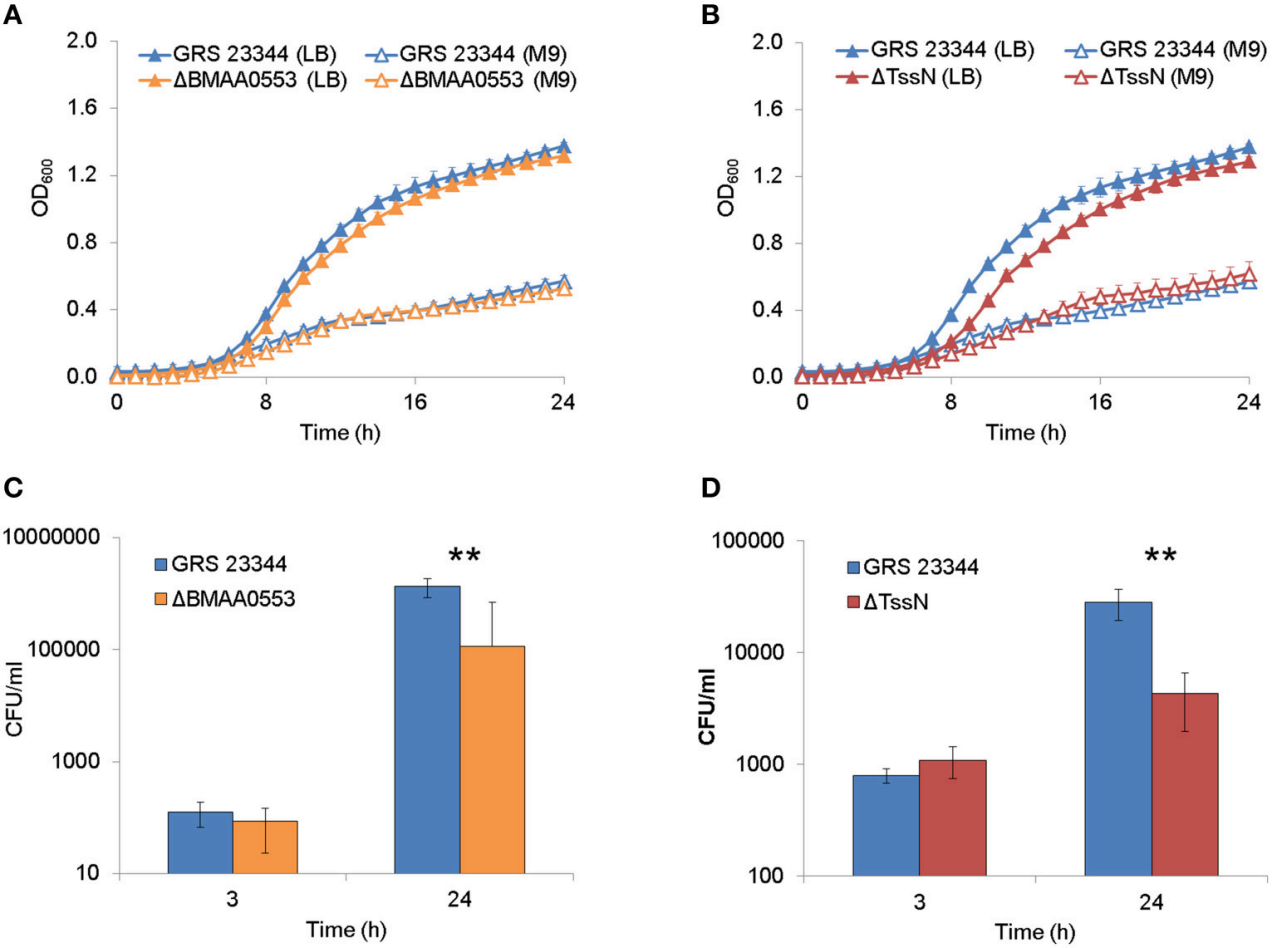
**Extracellular and intracellular growth**. Bacterial growth, as measured by optical density at 600 nm (OD_600_), in nutrient-rich LB media +4% glycerol (LB) and nutrient-limited minimal media (M9) are shown over 24 h for deletion strains ΔBMAA0553 **(A)** and ΔTssN **(B)** compared with the parental strain GRS 23344. Bacterial growth in RAW 264.7 macrophage-like cells is shown after 3 and 24-h incubation for GRS 23344 and ΔBMAA0553 at an MOI of 4 **(C)**, and GRS 23344 and ΔTssN at an MOI of 8 **(D)**. Growth was measured in terms of colony forming unit (CFU) recovery using gentamicin protection assays. Error bars represent standard errors from mean values determined from 2 to 4 replicates/assay. An “^**^” corresponds to a significant difference at *p* < 0.001.

ΔBMAA0553 and ΔTssN deletion strains were analyzed for any growth defects in LB + 4% glycerol or M9 broth cultures by OD measurements (Figures 1A,B). We used a logistic growth equation to fit the data as a function of maximum density, lag time, and maximum growth rate (Zweitering et al., 1990). For growth in LB media, we found that ΔBMAA0553 did not differ significantly from GRS 23344 in either maximum density, lag time or maximum growth rate, while ΔTssN showed a modest, but statistically significantly difference from GRS 23344 in lag time only, at 7.39 and 6.35 h, respectively (*p* < 0.001). For growth in M9 media, we observed no differences between GRS 23344 and either ΔBMAA0553 or ΔTssN (Figure 1B).

Next, the ability of ΔBMAA0553 and ΔTssN to replicate in host cells was measured. A macrophage-like cell line (RAW 264.7 cells) was infected and subsequently treated with gentamicin to kill extracellular bacilli. Figures 1C,D shows that the CFU values recovered at 24 h for both mutants were approximately 10-fold less than the parental strain, GRS 23344 (*p* < 0.001 for both mutants). However, no defects in uptake were observed for either of the mutants, as indicated by the 3-h time point.

### Phagosomal escape and actin tail formation

**Figure 2:**
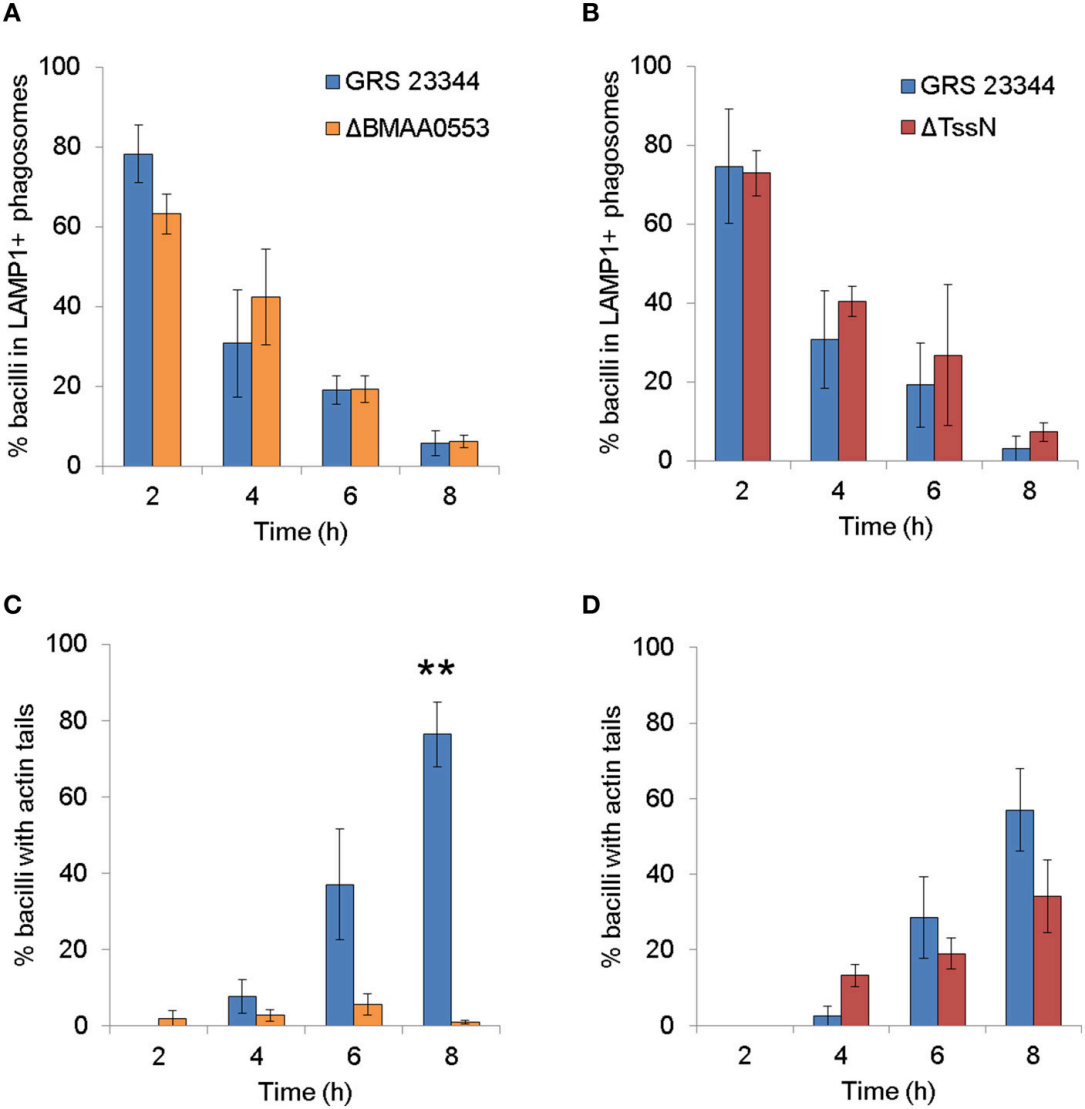
**Confocal analysis of the interaction of ***Burkholderia mallei*** (***Bm***) with RAW 264.7 cells**. Macrophage-like cells were incubated with GRS 23344 (*blue*), ΔBMAA0553 (*orange*), or ΔTssN (*red*), stained and then visualized by confocal microscopy. The colocalization of the respective *Bm* strains with LAMP-1 **(A,B)** or actin tails **(C,D)** was quantified at the times indicated postinfection. The graphs are based on three independent experiments. The number of bacilli counted was 686 for **(A)**, 724 for **(B)**, 673 for **(C)**, and 818 for **(D)**. The columns represent the mean, and bars represent the standard errors. An “^**^” indicates a significant difference at *p* < 0.001.

We investigated the ability of ΔBMAA0553 and ΔTssN to escape the phagosome in infected RAW 264.7 cells by colocalization of the mutants with LAMP-1 (Sajjan et al., 2006), a protein which delineates vacuoles of the endocytic pathway. Figures 2A,B shows that GRS 23344, ΔBMAA0553, and ΔTssN were localized in the LAMP-1 positive phagosome at 2 h postinfection, but localization in these compartments decreased over time, suggesting the parental strain and mutants escaped from the degradative pathway. The rate at which all three strains escaped was similar, indicating that ΔBMAA0553 and ΔTssN were unaltered in their ability to escape the phagosome.

We also examined the ability of ΔBMAA0553 and ΔTssN to form actin tails. Both the parental strain GRS 23344 and ΔTssN exhibited actin tails at the bacterial poles beginning at 4 h postinfection and increased in the number of bacteria with tails to 8 h (Figures 2C,D). This confirms that ΔTssN has the ability to escape and polymerize actin. In contrast, no significant actin tail formation, or nucleation was seen at the poles of ΔBMAA0553 bacteria compared to GRS 23344 at 8 h (*p* < 0.01). Thus, ΔTssN is motile in the cytoplasm, whereas ΔBMAA0553 was defective in actin tail formation.

### Multinucleated giant cell formation

**Figure 3:**
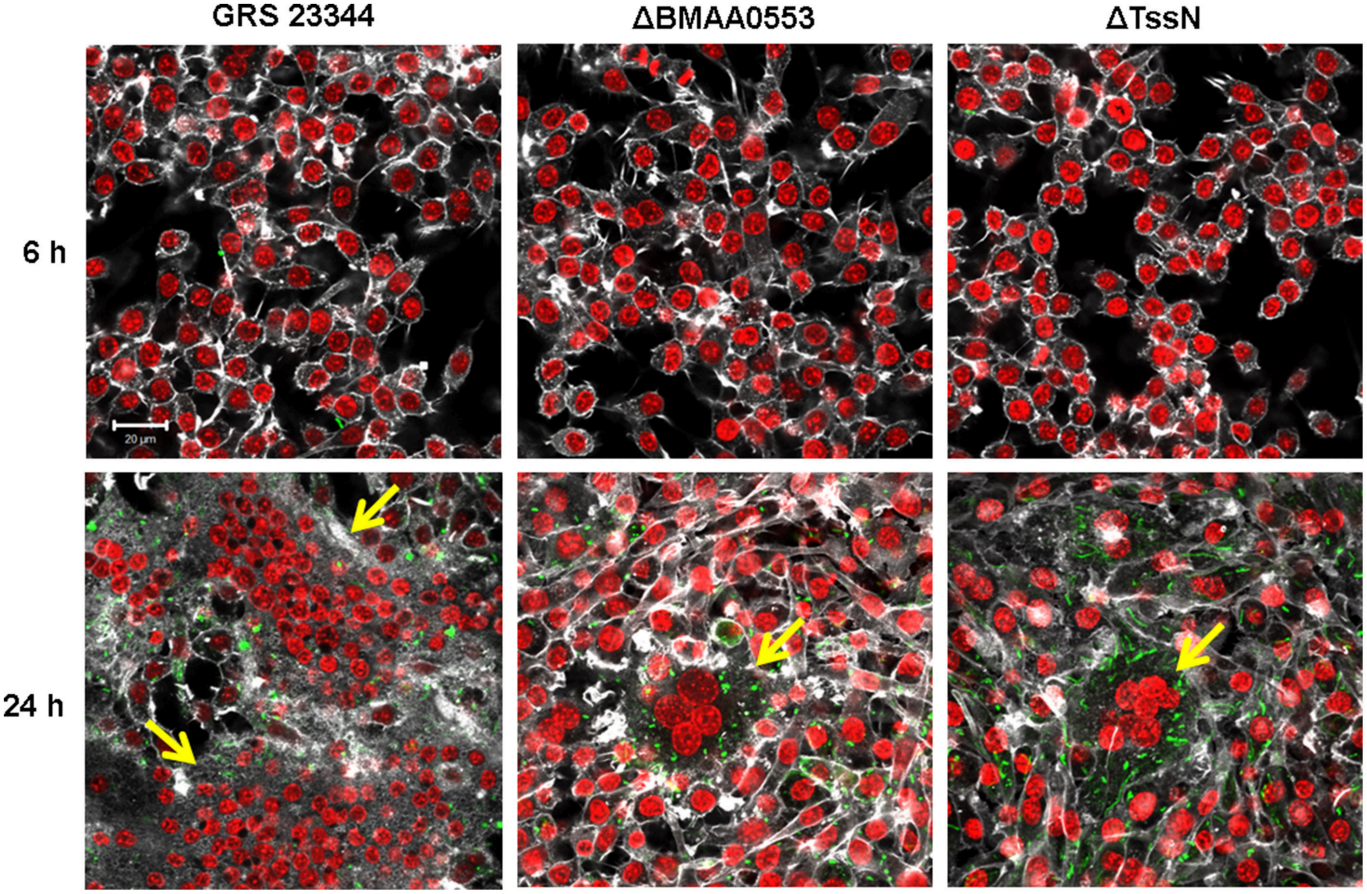
**Multinucleated giant cell (MNGC) formation in macrophage-like cells**. RAW 264.7 cells were incubated with GRS 23344 (*left*), ΔBMAA0553 (*middle*), or ΔTssN (*right*) at an MOI of 1 for 6 h (*top*) and 24 h (*bottom*) before being stained for actin (white), macrophage nuclei (red) and *Bm* (green) and then visualized by confocal microscopy. The yellow arrows indicate the presence of a MNGC. Images are representative of three separate experiments. Scale bar = 20 μm.

We examined the ability of ΔBMAA0553 and ΔTssN mutants to induce MNGC formation after infection (Harley et al., 1998; Brett et al., 2008). RAW 264.7 cells were infected with GRS 23344, ΔBMAA0553 or ΔTssN at a MOI of 1, immunostained, and imaged with a confocal microscope (Figure 3). At 6 h post infection, no MNGC was seen with infection of GRS 23344 or either mutant. At 24 h, infection with GRS 23344 at an MOI of 1 readily induced MNGC formation that incorporated between 50 and 200 nuclei/cell (Figure 3). In contrast, we observed mostly mononucleated cells with some smaller MNGC (3–10 nuclei) interspersed in monolayers infected with ΔBMAA0553 or ΔTssN at 24 h. We therefore also infected cells at a higher MOI of 20 and examined MNGC formation at 24 h (data not shown). At this higher MOI, we observed pyknotic nuclei and denuded plasma membranes, in a manner consistent with having formed MNGC first, and subsequently undergoing cell death, for GRS 23344 and ΔTssN, but not for ΔBMAA0553. Thus, ΔTssN still induced MNGC formation although at a slower rate than the parental strain whereas ΔBMAA0553 likely is not able to induce MNGC formation.

### Virulence and immune responses

**Figure 4:**
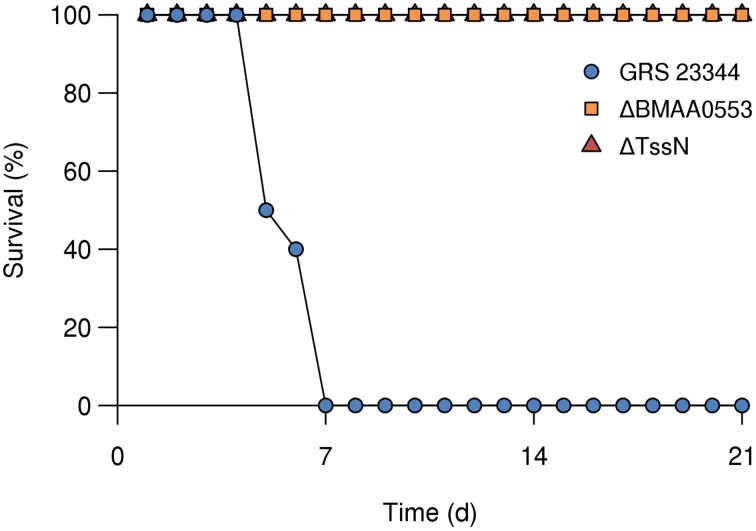
**Survival of BALB/c mice after aerosol exposure**. Groups of mice (*n* = 10) were challenged by aerosol exposure with GRS 23344 (blue), ΔBMAA0553 (orange), or ΔTssN (red), and survival was measured over 21 days. The measured inhaled dose was 4.7 × 10^4^, 3.3 × 10^4^, and 2.1 × 10^5^ CFU, for GRS 23344, ΔBMAA0553, and ΔTssN, respectively.

We measured the virulence of these mutant strains in a small particle aerosol challenge model of glanders where BALB/c mice were exposed to *Bm* in an aerosolized form. We first showed that GRS 23344 was able to cause infection in this model. As shown in Figure 4, when mice inhaled a measured dose of 4.7 × 10^4^ CFU of the parent strain, all mice succumbed to infection by day 7. Based on estimates that ~10^3^ CFUs of aerosolized *Bm* ATCC 23344 is necessary for an aerosol LD_50_ with BALB/c mice (Jeddeloh et al., 2003; Lever et al., 2003), these animals received the equivalent of 50 LD_50_.

In order to assess the virulence of ΔBMAA0553 and ΔTssN, we carried out a preliminary study where we aerosol challenged BALB/c mice (*n* = 10) with 8.9 × 10^3^ CFUs (the equivalent of 9 times the LD_50_ of ATCC 23344) of ΔTssN and observed them for 21 days. All mice survived the aerosol challenge over that time span and never displayed any clinical signs of disease. At *day 21* no *Bm* could be recovered from the spleens of the challenged mice. We repeated the experiment at a higher dose of 2.1 × 10^5^ CFUs of ΔTssN (equivalent to 200 LD_50_ of ATCC 23344) with similar results (Figure 4). We then tested the virulence of ΔBMAA0553 using aerosol challenge with a measured dose of 3.3 × 10^4^ CFUs (equivalent to 33 LD_50_ of ATCC 23344), and found that all mice survived over the study period of 21 days and were symptom free. These results show that the ΔBMAA0553 and ΔTssN strains are severely attenuated in the aerosol challenge model.

### Antibody responses and cytokine profiles

All mice in the ΔTssN or ΔBMAA0553 challenge survived through the 21-day study period. Using an ELISA against irradiated *Bm* of the respective challenge strain, we analyzed the overall IgG antibody response and the IgG subclass (IgG1 and IgG2a) response from serum samples of all challenged mice and compared the results to those from naïve mice. We found that all survivors showed a modest but highly variable IgG response, with titers of 2972 and 2425 for the ΔBMAA0553 and ΔTssN challenges, respectively (Table 1). ΔTssN challenges induced a T helper (Th)1-biased response, with an IgG2a-to-IgG1 ratio of 5.3. ΔBMAA0553, by contrast, induced a more balanced Th1/Th2 response with an IgG2a-to-IgG1 ratio of 1.1, attributed largely to an increased IgG1 response. Naïve mice did not show substantial antigen-specific antibody titers.

**Table 1:** **Antibody responses to ΔBMAA0553 and ΔTssN after aerosol exposure in BALB/c mice**.

**Mouse Group (*n*)**	Treatment (LD_50_)^*^	**Antibody titer (SD)**			**Ratio**
		**IgG**	**IgG1**	**IgG2a**		**IgG2a/IgG1**
Naïve (10)	–	<50 (0)	<50 (0)	<50 (0)	–
ΔBMAA0553 (10)	40	2972 (4972)	1213 (1956)	1131 (1013)	1.1
ΔTssN (5)	200	2425 (5110)	152 (325)	800 (3175)	5.3

* *50% Lethal dose (LD_50_) values were calculated based on an equivalent dose of Bm ATCC 23344 strain*.

**Figure 5:**
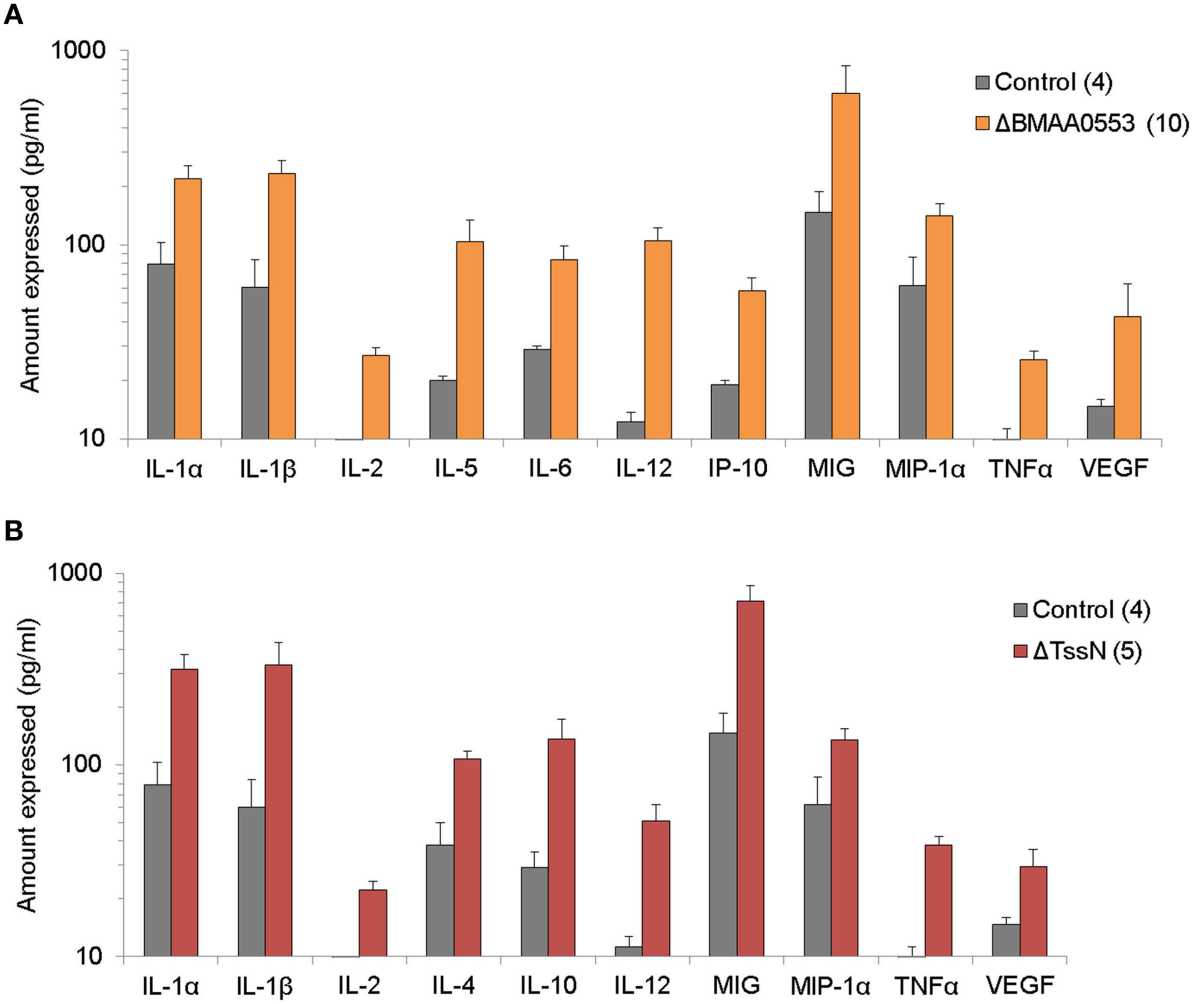
**Cytokine expression profile**. The levels of 20 cytokines/chemokines present in spleen extract supernatants were examined in mice after aerosol exposure to ΔBMA0553 **(A)** or ΔTssN **(B)** and compared to control mice 21 days postinfection. Only the expression levels of cytokines/chemokines that were at least two-fold above that of naïve mice are shown. All differences shown are statistically significant at *p* < 0.001.

We examined the protein levels of 20 cytokine/chemokines in the spleen extract supernatant of surviving mice prepared after the 21-day study period. We considered cytokine/chemokines that were elevated by at least two-fold relative to levels found in naïve BALB/c mice as significant. In spleen extracts from mice challenged with ΔBMAA0553, we found 11 cytokines that were elevated (Figure 5A), including IL-1α, IL-1β, IL-2, IL-5, IL-6, IL-12, IP-10, MIG, MIP-1α, TNF-α, and VEGF. In spleen extracts from mice that survived the higher dose challenge of ΔTssN, we found 10 cytokines that were elevated (Figure 5B; IL-1α, IL-1β, IL-2, IL-4, IL-10, IL-12, MIG, MIP-1α, TNF-α, and VEGF). In both cases, these cytokines represent a mixture of proinflammatory and inflammatory cytokines that included chemoattractants for cells of the immune system. In both ΔBMAA0553 and ΔTssN challenges, IFNγ levels were increased, but not to the two-fold threshold (data not shown). The elevated cytokine levels in these challenged mice 21 days postinfection show that an innate or cellular immune response appears to have been mounted in response to ΔBMAA0553 and ΔTssN aerosol exposure.

### Bacterial dissemination

**Figure 6:**
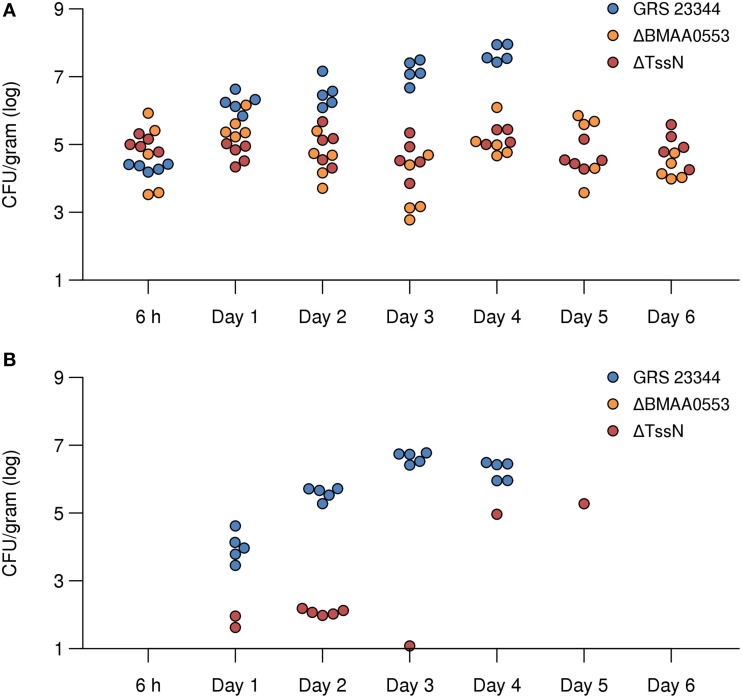
**Bacterial dissemination after infection in the lung and spleen in mice**. Recovered CFUs from the lung **(A)** and spleen **(B)** are shown for time points ranging from 6 h to 6 days after aerosol exposure with parental strain GRS 23344 (blue), or the deletion strains ΔBMAA0553 (orange), and ΔTssN (red). In mice challenged with ΔBMAA0553, none had >10 CFUs recovered from the spleen.

To determine the fate of the mutant strains, a dissemination experiment was performed to compare the mutant ΔBMAA0553 and ΔTssN strains with the parental GRS 23344 strain after aerosol exposure (*n* = 5 for each time point). As shown in Figure 6A, when BALB/c mice were challenged with GRS 23344 (estimated inhaled dose of 4.4 × 10^4^ CFU), the bacterial numbers quickly increase within the lungs by *day 1*. In addition, *Bm* was detected in the spleens for all mice by *day 1*, and the numbers increase thereafter (Figure 6B). By *day 4*, the remaining mice were either moribund (used for CFU recovery) or had succumbed to infection. Therefore, no mice were available for sampling past this time point.

In contrast, none of the mice aerosol challenged with either ΔBMAA0553 (estimated inhaled dose of 4.4 × 104 CFUs) or ΔTssN (estimated inhaled dose of 1.6 × 10^5^ CFUs) showed an increase in the recovery of CFUs from the lungs (Figure 6A) but CFUs were still recovered from the organs during the course of the study. In addition, few CFUs were recovered from the spleens (Figure 6B). Overall, none of the mice challenged with ΔTssN or ΔBMAA0553 displayed any outward clinical signs of disease. These results demonstrate a defect of these two mutant strains to replicate *in vivo* and to successfully disseminate from the lungs in this mouse inhalation model of infection.

### Live-attenuated vaccine

LAVs provide a potent means for inducing a durable and protective immune response by mimicking the infection of more virulent strains. Given that ΔTssN showed attenuated pathogenic phenotypes *in vitro* and was effectively tolerated *in vivo*, we sought to evaluate its potential as a live-attenuated glanders vaccine. Because of the modest antibody responses measured in the initial aerosol exposure experiments using ΔTssN, we used a prime-boost vaccination strategy to boost adaptive immune responses to ΔTssN before aerosol challenge with ATCC 23344.

We vaccinated mice (*n* = 15) two times by aerosol with the mutant strain ΔTssN, whereas a second set of mice (*n* = 10) was retained as naïve controls and remained untreated. The inhaled dose of the first vaccination with ΔTssN was calculated to be 1.3 × 10^5^ CFU. After a period of 21 days, mice showed no sign of adverse events associated with the infection and were vaccinated a second time by aerosol. The second inhaled dose was calculated to be 2.3 × 10^4^ CFU. Mice were followed for 21 days and again showed no adverse signs of infection. At this time, vaccinated and naïve control mice were challenged by aerosol with a high dose of *Bm* ATCC 23344 strain (4.3 × 10^4^ CFUs, or 43 LD_50_).

**Figure 7:**
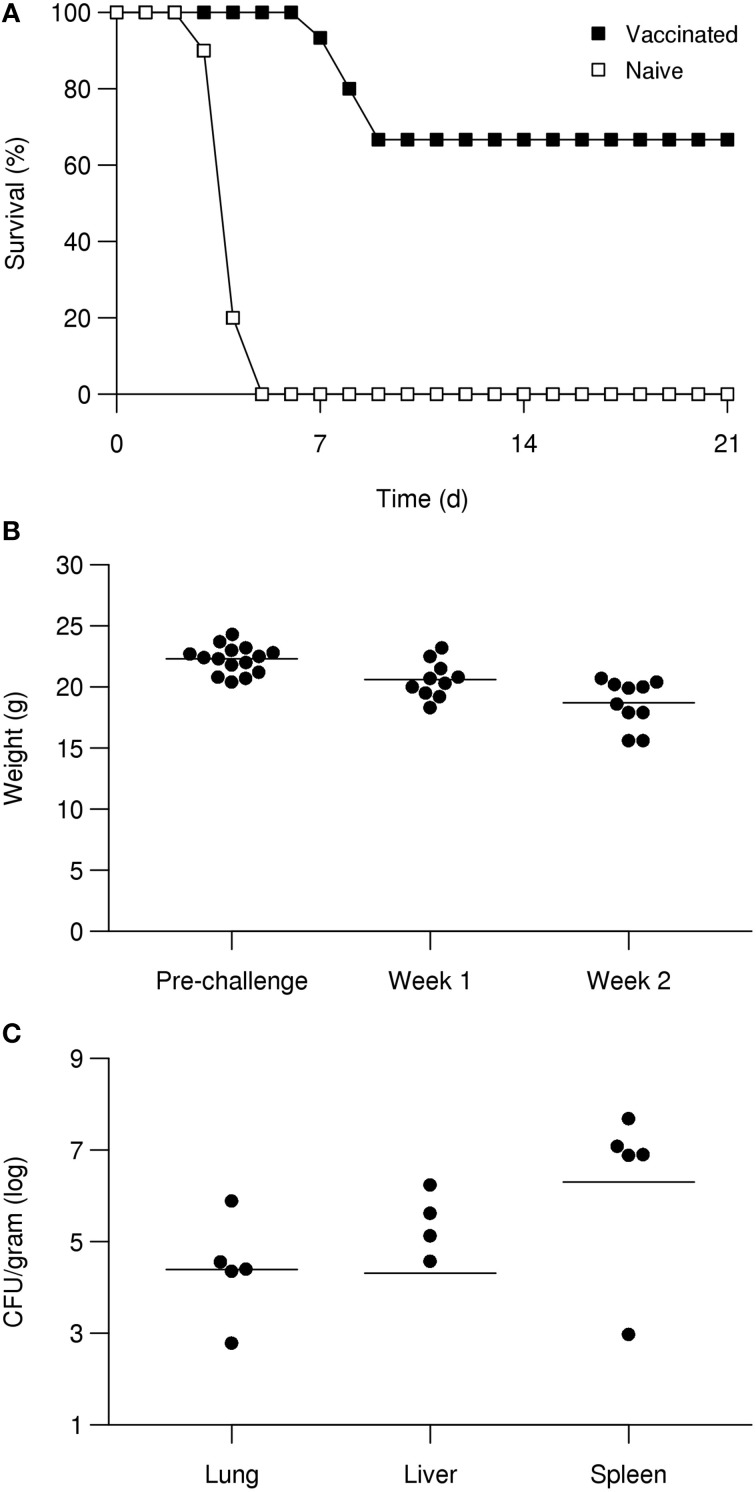
**ΔTssN vaccine efficacy against aerosol challenge with high-dose ATCC 23344 in BALB/c mice**. Mice were vaccinated with ΔTssN via aerosol delivery on *day* 0 and 21 and challenged along with naïve mice via aerosol exposure on *day* 42. Survival curves of naïve and vaccinated mice after aerosol challenge with 43 LD_50_ of *Bm* ATCC 23344 over 21 days **(A)**. Body weights of vaccinated surviving mice at prechallenge, 2 week postchallenge, and 3 week postchallenge **(B)**. Recovered CFUs from five of the vaccinated surviving mice at 21 days postchallenge from the lung, liver, and spleen **(C)**.

As shown in Figure 7A, all naïve mice succumbed to infection by *day 5*. In contrast, 67% of the vaccinated mice survived to *day 21*. When comparing survival rates between the two groups, the difference was significant (*P* < 0.001). In addition, for those vaccinated mice (*n* = 5) that did succumb to challenge, the mean time to death was 8.2 days which was significantly longer (*P* < 10^−4^) than the mean time to death of 4.1 days for the control group.

Despite the increased survival rate for vaccinated mice, we observed that survivors experienced a significant weight loss after challenge (Figure 7B). At 2 week postchallenge, vaccinated animal weights decreased by a mean of 1.7 g (*P* < 0.05) from the mean weight before challenge. At 3 week postchallenge, vaccinated animal weights decreased by a mean of 3.6 g (*P* < 10^−4^) from the mean weight before challenge. For the ten mice that did survive the challenge, five were examined by histopathology and the remaining five mice were used to determine if sterile immunity was achieved. As shown in Figure 7C, we were able to recover *Bm* CFUs from a majority of organs tested (lung, liver, and spleen) for all five mice.

### Vaccine-induced immune response

Table 2 shows the antibody response to *Bm* ATCC 23344 21 days after the prime vaccination and 21 days after the boost vaccination with ΔTssN. An ELISA using heat-inactivated *Bm* ATCC 23344 showed antibody responses that were comparable to the modest responses that we previously observed in the aerosol exposure experiments with ΔTssN (Table 1). At 21 days after the prime vaccination, we saw an average IgG titer of 3658, with average IgG subclass titers of 1051 and 3185, for IgG1 and IgG2a, respectively. Surprisingly, at 21 days after the boost vaccination with ΔTssN, we observed that antibody titers were lower than after the prime vaccination, with average IgG titers of 400 and average IgG1 and IgG2a titers of 87 and 303, respectively.

**Table 2:** **Antibody response to ΔTssN vaccination in BALB/c mice**.

	**Treatment**	**Antibody titer (SD)**			**Ratio**
**Mouse group**		**(CFUs)**	**IgG**	**IgG1**		**IgG2a**	**IgG1/IgG2a**
Naïve	–	<50 (0)	<50 (0)	<50 (0)	–
ΔTssN, Prime (*day 23*)	1.25 × 10^5^	3658 (6212)	1051 (6812)	3185 (6365)	3.0
ΔTssN, Boost (*day 44*)	2.28 × 10^4^	400 (219)	87 (61)	303 (261)	3.5

Furthermore, we examined cytokine expression in supernatant following *in vitro* stimulation by irradiated *Bm* of splenocytes prepared from mice after the prime and boost vaccinations (Supplementary Figure S1). Like the antibody responses, we did not observe an overall increase of cytokine expression after the boost vaccination in vaccinated mice. Overall, these results show only modest antibody and cellular responses induced by the LAV, suggesting poor immunogenicity with respect to the adaptive immune response.

### Histopathology of vaccinated mice

**Figure 8:**
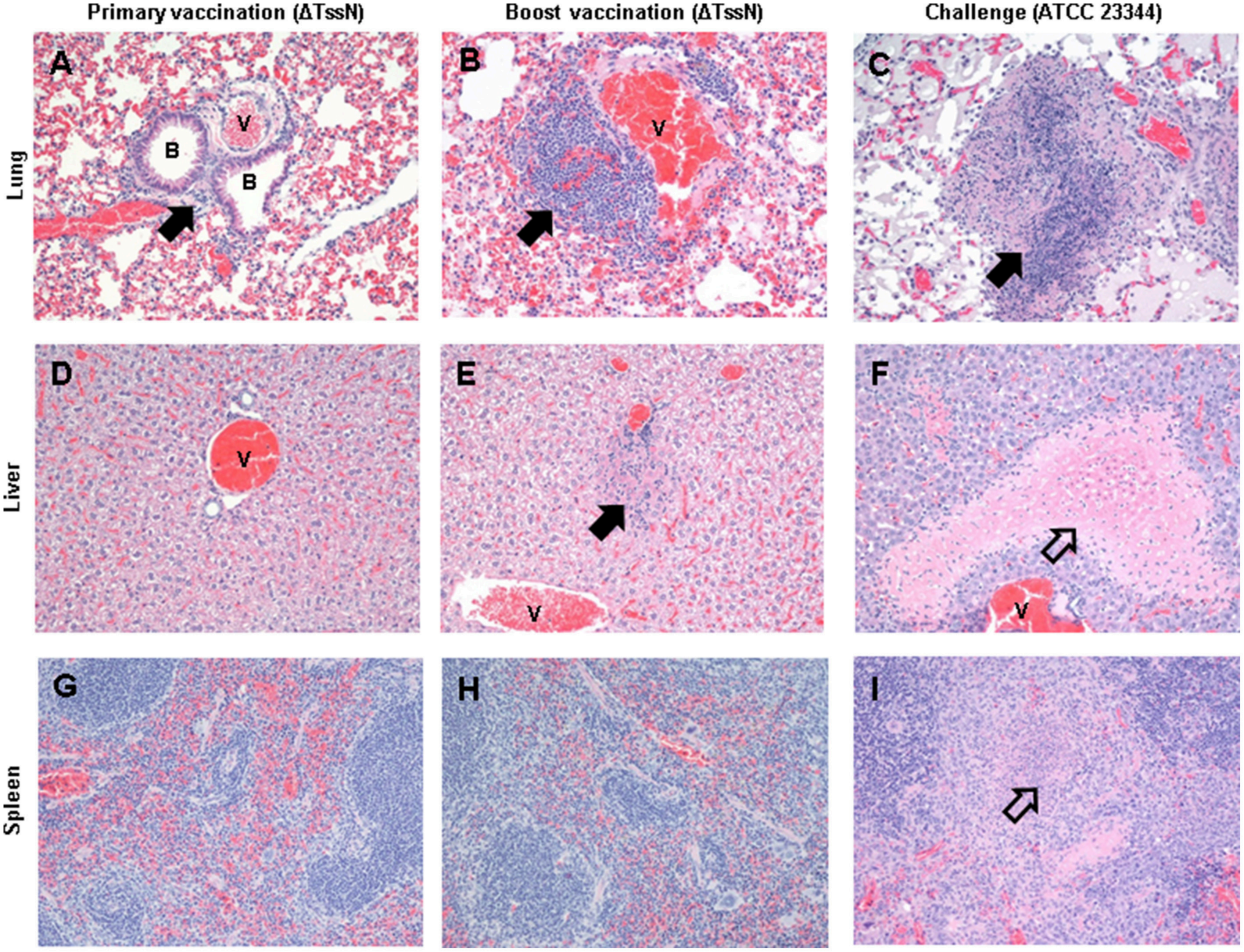
**Histopathology of vaccinated and challenged mice**. Histopathology of the lung **(A–C)**, liver **(D–F)**, and spleen **(G–I)** for mice 21 days after primary and boost vaccinations with ΔTssN, and after challenge with ATCC 23344. Sites of inflammation (filled arrow) and necrosis (hollow arrow) are noted, as well as relevant bronchioles (B) and blood vessels (V). All images are at × 200 magnification.

We characterized the histopathology of the lung, liver, and spleen of vaccinated and challenged mice. After the first vaccination with ΔTssN, lungs of all mice had minimal nonspecific inflammation. Low to moderate numbers of plasma cells were observed in thoracic lymph nodes, indicative of humoral immune stimulation with subsequent antibody production. Similarly, for mice that received the boost vaccination, the lungs had minimal to mild inflammation, and the pulmonary infiltrates of two mice had plasma cells, suggesting prior humoral stimulation. Lungs of all surviving mice at 21 days post challenge demonstrated signs of pneumonia characterized by effacement of the parenchyma by necrotic cellular debris, pyogranulomatous inflammation, many foamy macrophages and scattered hyperplastic type II pneumocytes. Representative lung sections are shown in Figures 8A–C, with low numbers of lymphocytes and macrophages surrounding bronchioles and blood vessels at 21 days after primary vaccination (Figure 8A), increased lymphocytes and macrophages at 21 days after the boost vaccination (Figure 8B), and necrosis with necrotic cellular debris and fibrin and edema in adjacent alveolar spaces in mice that survived 21 days after challenge (Figure 8C).

The livers of mice after the prime and boost vaccinations showed minimal to mild inflammation, whereas the livers of the vaccinated mice that survived WT challenge experienced extensive necrosis. Figures 8D–F shows representative liver sections showing minimal inflammation at 21 days after primary vaccination (Figure 8D), mild hepatocellular degeneration with infiltration by low numbers of lymphocytes, macrophages and neutrophils after the boost vaccination (Figure 8E), and a large focally extensive area of coagulative necrosis at 21 days after challenge by ATCC 23344 (Figure 8F). The most common splenic lesion in all mice was minimal to mild expansion of the white pulp by lymphoid hyperplasia with variable numbers of plasma cells within the white and red pulp (Figures 8G–I). However, two mice from the challenged group had necrosis of the white and red pulp and infiltration of neutrophils in one case (Figure 8I) and primarily neutrophilic and histiocytic inflammation in the other case. All of the surviving mice had plasma cells present within their thoracic lymph nodes (data not shown), suggesting that exposure to the WT strain may have provided a boost to immunity.

## Discussion

In the present study, we constructed deletion mutants for *BMAA0553* and *tssN*, characterized their pathogenic phenotypes *in vitro* and *in vivo*, and evaluated the potential of ΔTssN as a LAV in a mouse model for inhalational glanders. *In vitro* growth assays in nutrient-limited M9 media revealed no difference between the parental *Bm* strain GRS 23344 and ΔBMAA0553 and ΔTssN mutants, suggesting that these genes do not play a major role in basic metabolism. Intracellular growth assays with macrophage-like cells showed that while both the parental strain and deletion mutants had comparable recovery at 3 h, both deletion mutants showed a ~10-fold reduction in growth at 24 h, indicating that the decreased intracellular survival is a result of phenotypic changes associated with the host cell environment and not host cell entry. Microscopy of infected macrophages revealed that both ΔBMAA0553 and ΔTssN were able to undergo phagosomal escape and that ΔBMAA0553, but not ΔTssN, showed a marked reduction in actin tail formation, indicating a defect in intracellular motility. Furthermore, ΔTssN displayed a marked delay in both the size and number of MNGCs formed *in vitro* relative to the parent GRS 23344 strain, while ΔBMAA0553 was almost entirely unable to form MNGCs.

Both ΔBMAA0553 and ΔTssN showed significant attenuation of virulence in BALB/c mice. Mice exposed to aerosol challenge with ΔBMAA0553 or ΔTssN, even at high doses, had 100% survival 21 days postinfection, compared to mice infected with GRS 23344, which died within 5 days. Both ΔBMAA0553 and ΔTssN persisted within the lungs following inhalation, and ΔTssN was recovered from spleens between *days 1–3* for some mice, showing that while these mutants are able to survive within the lung, they are largely unable to establish a systemic infection. Analysis of the antibody response of infected mice revealed a Th1-biased antibody response following ΔTssN infection and a balanced Th1/Th2 response to ΔBMAA0553. These results reflect previous findings that show that *Bm* live infection induces a Th1-biased response, whereas nonviable *Bm* induces a balanced Th1/Th2 response (Amemiya et al., 2002; Ulrich et al., 2005) and supports our findings that ΔTssN is able to establish at least a mild infection whereas ΔBMAA0553 may be completely avirulent. Cytokine analysis at 21 days postinfection revealed numerous elevated pro-inflammatory and inflammatory cytokines, including IL-2, IL-12, TNF-α, and VEGF. A number of these cytokines are markers for activated macrophages (Martinez and Gordon, 2014), suggesting engagement of the innate immune response. Furthermore, the elevated cytokine levels 3 week postinfection shows that despite the high degree of attenuation, both strains are able to induce a prolonged innate or cellular immune response. Finally, many of these cytokines have been previously associated with a protective immune response in *Bm* infection, including IL-2 (Whitlock et al., 2011), IL-12 (Amemiya et al., 2006), and TNF-α (Whitlock et al., 2008).

### *Burkholderia* virulence factor mechanisms

A number of virulence factors have been previously characterized in *Bm* and *Bp*, including VirAG, a two-component regulatory system shown to be essential for virulence in *Bm* (Schell et al., 2007). Among several virulence gene clusters transcriptionally activated by VirAG is a cluster of motility-associated genes, *BMAA0747-BMAA0751*, which includes the genes *bimA, bimB, bimC*, and *bimE*. Mutations to each of these genes abolish or modify actin-based tail formation (Stevens et al., 2005b; Schell et al., 2007; Benanti et al., 2015; Lu et al., 2015), demonstrating that the interaction of multiple genes is involved in *Bm* intracellular motility. Other studies have shown that *bimA* mutation does not affect host cell entry or phagosomal escape (Stevens et al., 2005b) but can modulate MNGC formation (Benanti et al., 2015). Although we found that deletion of *BMAA0553* abolished actin tail formation, no previously characterized *Bm* gene associated with actin-based motility was essential for MNGC formation and *in vivo* infectivity, suggesting that the role of *BMAA0553* in virulence extends beyond motility alone.

A second gene cluster activated by VirAG is *BMAA0744*-*BMAA0727* (including *tssN*), which encodes components of a type 6 secretion system (Schell et al., 2007). Overexpression of VirAG leads to the secretion of virulence factors, such as Hcp1 and VgrG1, and experiments in *Bp* have indicated that these genes are also involved in MNGC formation (Burtnick et al., 2011; Toesca et al., 2014). Although *tssN* has not been previously characterized, neighboring, co-expressed, *BMAA0729* (*tssM*) has been extensively studied. *tssM* is expressed in *Bm* and *Bp* shortly after uptake by macrophage cells (Shalom et al., 2007; Shanks et al., 2009), has deubiquitinase activity (Shanks et al., 2009; Burtnick et al., 2014), and is responsible for suppression of host innate immunity (Tan et al., 2010). However, *tssM* has no effect on intracellular survival, is not essential for MNGC formation and is not required for virulence in hamsters (Shanks et al., 2009). We found that, unlike *tssM* (Shalom et al., 2007; Shanks et al., 2009), *tssN* deletion was associated with decreased intracellular survival, reduced or delayed MNGC formation, and loss of virulence.

Previous bioinformatics analyses indicate that *tssN* is involved in host ubiquitination and intracellular signaling and that *BMAA0553* encodes a putative serine/threonine phosphatase that plays a role in signaling, adhesion, and actin cytoskeletal rearrangement (Memiševic et al., 2014, 2015). Association of *BMAA0553* with MNGC formation is particularly intriguing because of its genomic localization far from the T6SS cluster, which is linked to MNGC formation (Toesca et al., 2014). Toesca et al. showed that the VgrG C-terminal domain is required for cell fusion, but they and others suggested that it might not be sufficient and that additional mechanisms may be involved (Stone et al., 2014; Toesca et al., 2014). The previously identified role of *BMAA0553* in interaction with host signaling pathways, adhesion, and actin cytoskeleton rearrangement (Memiševic et al., 2014, 2015) might be interesting for further study within the context of MNGC formation, as suggested by our observation of the loss of this phenotype in ΔBMAA0553.

### LAV and immune characteristics of ΔTssN

Given the ability of ΔTssN to display several pathogenic phenotypes, such as establishing a lung infection, macrophage entry, phagosomal escape, intracellular motility, and limited MNGC formation and as well as the previous association of *tssN* with the type 6 secretion system (Schell et al., 2007), we investigated the use of ΔTssN as a glanders LAV. Aerosolized prime-boost vaccination with ΔTssN showed 67% efficacy in protecting against high-dose (>50 LD_50_) aerosolized challenge with *Bm* ATCC 23344 by 21 days postinfection. Subsequent analysis showed that despite the protection achieved from acute *Bm* infection, all surviving mice had detectable bacteria in the lungs, spleen, and liver, showing that sterile immunity was not achieved.

Analysis of antibody and cytokine responses in immunized mice revealed a modest Th1-biased antibody response that was comparable with other *Bm* LAVs (Ulrich et al., 2005) but were not enhanced during boost immunizations. Stimulation of splenocytes from vaccinated mice with irradiated ΔTssN and ATCC 23344 showed robust cytokine responses and the cytokine expression profile for both strains was virtually identical, underscoring their antigenic similarity. However, we found no change in the cytokine response in spleen samples from mice after prime and boost immunizations, suggesting that the protective immune response induced by ΔTssN was primarily an innate response. Interestingly, a previous study (Fernandes et al., 2007) has shown substantial protective effects of adjuvant alone to aerosol challenge, even several weeks after immunization, indicating that innate immunity can play a significant role in protection in aerosol exposure. Furthermore, a study with aerosol exposure with the intracellular bacterial pathogen *Francisella tularensis* has shown delayed or prolonged innate responses that are not seen in intradermal or subcutaneous routes of infection (Conlan et al., 2002).

Although it is difficult to directly compare efficacy results across studies due the variety of immunization and challenge models, the 67% protection over 21 days after a high-dose (43 LD_50_) challenge in BALB/c mice achieved by ΔTssN vaccination observed in this study is comparable with the protection achieved by other glanders vaccine candidates to aerosol challenge. Ulrich et al. (2005) using a genetically engineered *Bm* auxotroph, saw 50% protection against low-dose (5 LD_50_) and 35% protection against high-dose (440 LD_50_) at 28 days. Fernandes et al. (2007) found 30% efficacy when vaccinating with irradiated *Bm* and no efficacy when vaccinating with *Bm* pilin protein in BALB/c mice after high-dose (22 LD_50_) challenge. Likewise, Torres et al. (2015), using a gold nanoparticle-based subunit vaccine, found no difference in protection between vaccinated and unvaccinated rhesus macaques after a low-dose (~1 LD_50_) aerosol exposure.

It is important to note that unlike intramuscular, intraperitoneal, or subcutaneous routes of administration which directly induce a systemic immune response, aerosol exposure is localized in the lung, and thus the most relevant immune responses are likely localized in the lung mucosal environment. However, directly measuring antibody, cytokine, or cellular responses in the lung using methods, such as alveolar lavage, is extremely difficult to perform, especially under containment conditions. Our use of serum antibodies and spleen extracts focuses on the systemic immune response and may only serve as a surrogate for the relevant immune environment in the lung mucosa.

Characterization of the ΔBMAA0553 and ΔTssN strains showed defects in intracellular growth and survival, associated with a loss of intracellular motility and delayed MNGC formation, respectively, and was found to have highly attenuated virulence *in vivo*. Vaccination with ΔTssN followed by high-dose aerosol challenge showed partial protection against death associated with acute infection, but failed to prevent the establishment of chronic infection. Our findings suggest that a persistent low-level infection of the lung by immunization of ΔTssN induced a delayed or prolonged innate response that was likely responsible for the observed protection. Such a response would explain the unusually low antibody response after boost immunization, as it would rapidly clear the LAV before a secondary antibody response could be mounted. Despite the protection achieved in this study, the modest antibody responses induced by ΔTssN limits its potential use as a vaccine candidate, and instead serves to highlight the role of innate and cellular immunity in mitigating acute infection in inhalational glanders.

## Author contributions

The experiments were conceived and designed by JB, SC, KA, JC, JR, and AW. The experiments were performed by JB, KA, JC, CC, RT, JD, and CK. The data were analyzed by JB, KA, JC, CW, JR, and NZ. The manuscript was written by JB, SC, KA, JC, and AW.

### Conflict of interest statement

The authors declare that the research was conducted in the absence of any commercial or financial relationships that could be construed as a potential conflict of interest.
