# Supplementary material for: Phenotypic Characterization of a Novel Virulence-Factor Deletion Strain of Burkholderia mallei That Provides Partial Protection against Inhalational Glanders in Mice
Source: Front Cell Infect Microbiol. 2016 Feb 26;6:21. doi: 10.3389/fcimb.2016.00021 (PMC4767903; doi:10.3389/fcimb.2016.00021)
Supplement: Supplementary file 1 [file Table1.DOCX]

**Supplementary Table S1. Strains and plasmids**

| ***E. coli* strains** | **Relevant characteristics** | **Reference** |
| --- | --- | --- |
| DH5α | routine cloning | NEB |
| OneShot Pir1 | routine cloning for R6Kγ ori vectors | Invitrogen |
| S17-1 λpir | for conjugal transfer to *B. mallei* | (Wang and Kushner, 1991) |
| ***Bm* strains** |  |  |
| FMH | wild-type, human isolate derived from ATCC 23344 | (Allwood et al., 2011) |
| GRS 23344 | ΔsacB sucrose-resistant derivative | (Schell et al., 2008) |
| ΔTssN | In-frame deletion of *tssN*, based on the parent strain GRS 23344 | This study |
| ΔBMAA0553 | In-frame deletion of *BMAA0553*, based on the parent strain GRS 23344 | This study |
| **Plasmids** |  |  |
| pJET1.2 | PCR cloning vector | Fermentas, Inc. |
| pWSK30 | low copy *E. coli* vector for cloning | (Wang and Kushner, 1991) |
| pMo130 | aphA (kanamycin resistant) sacB (sucrose sensitive) | (Hamad et al., 2009) |
| pMo130-ΔTssN | vector containing in-frame deletion of *tssN* | This study |
| pMo130-ΔBPSS0403 | vector containing in-frame deletion of *BPSS0403* (*Bp* analog to *BMAA0553*) | This study |
