# Supplementary material for: Phenotypic Characterization of a Novel Virulence-Factor Deletion Strain of Burkholderia mallei That Provides Partial Protection against Inhalational Glanders in Mice
Source: Front Cell Infect Microbiol. 2016 Feb 26;6:21. doi: 10.3389/fcimb.2016.00021 (PMC4767903; doi:10.3389/fcimb.2016.00021)
Supplement: Supplementary file 2 [file Table2.DOCX]

**Supplementary Table S2. Primers**

Cloning *tssN* 5’ CAAGGCGATGCGATAGAACTC

Cloning *tssN* 3’ GCCCTCCGCGCCATCCACTGC

Delete *tssN* 5’ CACGCGCCGAAGCGGTGCGACGGC

Delete *tssN* 3’ tgatgtcatgattgacacccaata

Screen *tssN* 5’ gactccggctggccctgtttgtag

Screen *tssN* 3’ ttccgttcatgttcaccctctgtc

Screen 0553 5’ caaccccaaatgactgcttcttcc

Screen 0553 3’ cgtcgcggcgccgtgataccagt

Cloning Bpm0403 5’ GCTAGCCGCTCGCAACCGTGAAACAC

Cloning Bpm0403 3’ GCTAGCTCATCAGGAAACCGAAGCTG
