# Supplementary material for: Phenotypic Characterization of a Novel Virulence-Factor Deletion Strain of Burkholderia mallei That Provides Partial Protection against Inhalational Glanders in Mice
Source: Front Cell Infect Microbiol. 2016 Feb 26;6:21. doi: 10.3389/fcimb.2016.00021 (PMC4767903; doi:10.3389/fcimb.2016.00021)
Supplement: Supplementary file 3 [file Table3.DOCX]

**Supplementary Table 3. Survival and TTD comparisons between mice challenged intranasally with Bm ATCC 23344 and GRS 23344**

| **Strain** | **Dose (CFUs)** | **Surviving/Total** | **Survival (%)** | **TTD (days)^a^** |
| --- | --- | --- | --- | --- |
| ATCC 23344 | 180 | 9/10 | 90 | 5.00 |
| GRS 23344 | 170 | 8/10 | 80 | 11.0 |
| ATCC 23344 | 1,800 | 5/10 | 50 | 6.20 |
| GRS 23344 | 1,700 | 8/10 | 80 | 8.00 |
| ATCC 23344 | 18,000 | 0/10 | 0 | 4.00 |
| GRS 23344 | 17,000 | 0/10 | 0 | 4.50 |
| ATCC 23344 | 180,000 | 0/10 | 0 | 4.00 |
| GRS 23344 | 170,000 | 0/10 | 0 | 4.10 |
| ATCC 23344 | 1,800,000 | 0/10 | 0 | 4.00 |
| GRS 23344 | 1,700,000 | 0/10 | 0 | 4.00 |

^a^ Average time-to-death
