# Supplementary figures and images for: Phenotypic Characterization of a Novel Virulence-Factor Deletion Strain of Burkholderia mallei That Provides Partial Protection against Inhalational Glanders in Mice
Source: Front Cell Infect Microbiol. 2016 Feb 26;6:21. doi: 10.3389/fcimb.2016.00021 (PMC4767903; doi:10.3389/fcimb.2016.00021)

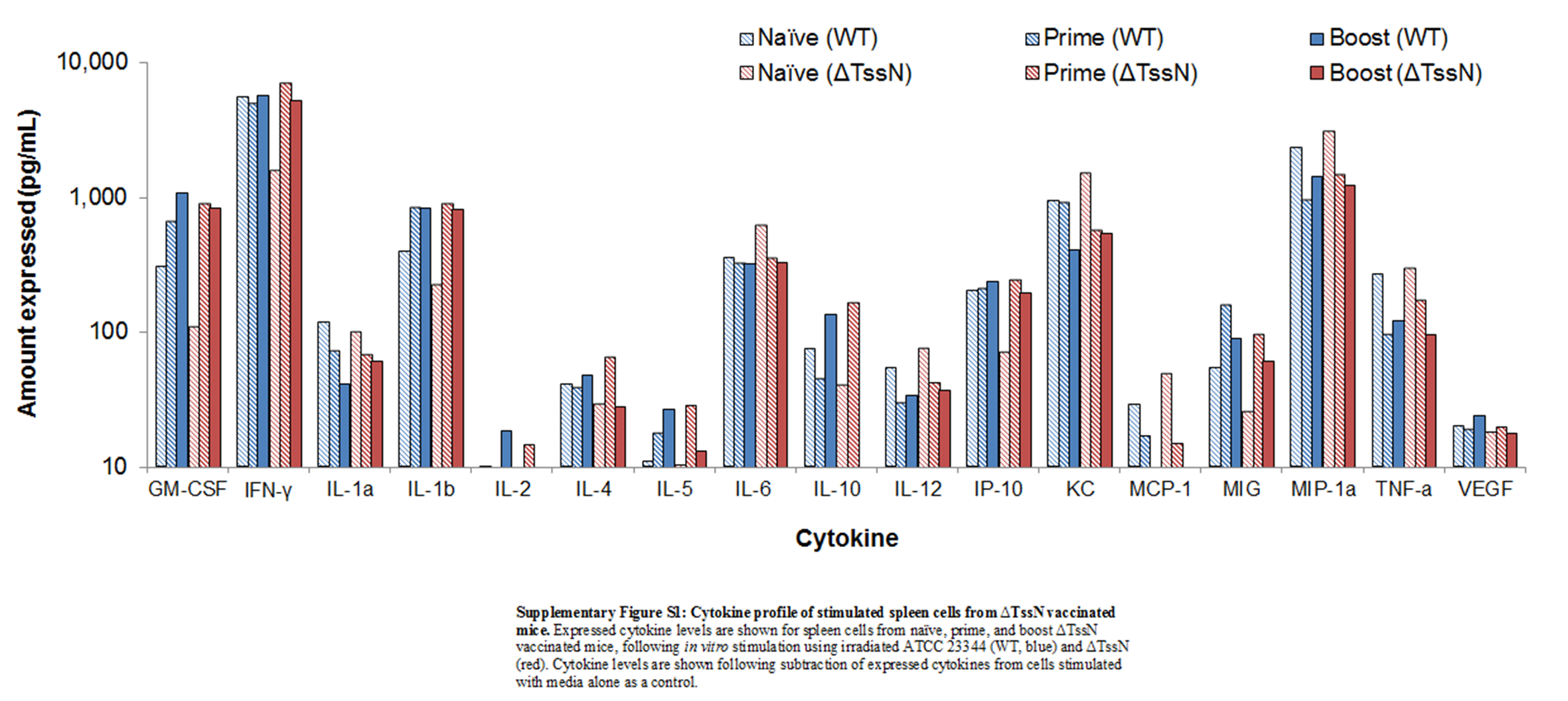

Supplement: Supplementary file 4 [file Image1.TIF]
